# Evaluating plasma antinuclear autoantibody profile as a prognostic biomarker in lymphoma

**DOI:** 10.1186/s12885-024-13198-2

**Published:** 2024-11-26

**Authors:** Cuiling Zheng, Ruyun Gao, Yanrong Wang, Xiaohong Han

**Affiliations:** 1 Department of Clinical Laboratory, National Cancer Center/National Clinical Research Center for Cancer/Cancer Hospital, Chinese Academy of Medical Sciences & Peking Union Medical College, No. 17 Panjiayuan Nanli, Chaoyang District, Beijing, 100021 China; 2 Department of Medical Oncology, National Cancer Center/National Clinical Research Center for Cancer/Cancer Hospital, Chinese Academy of Medical Sciences & Peking Union Medical College, Beijing Key Laboratory of Clinical Study On Anticancer Molecular Targeted Drugs, No. 17 Panjiayuan Nanli, Chaoyang District, Beijing, 100021 China; 3 Clinical Pharmacology Research Center, Peking Union Medical College Hospital, State Key Laboratory of Complex Severe and Rare Diseases, NMPA Key Laboratory for Clinical Research and Evaluation of Drug, Beijing Key Laboratory of Clinical PK and PD Investigation for Innovative Drugs, Chinese Academy of Medical Sciences and Peking Union Medical College, No.1 Shuaifuyuan, Dongcheng District, Beijing, 100730 China

**Keywords:** Lymphoma, Biomarker, Autoantibodies, Antinuclear antibodies

## Abstract

**Background:**

Research on the antinuclear antibodies (ANA) profile across different pathological subtypes of lymphoma was limited. Our study aimed to assess ANA profile and investigate its potential prognostic value in lymphoma.

**Method:**

We collected plasma samples from 139 lymphoma patients and analyzed the expression of plasma ANA, SSA, and SSB using the enzyme-linked immunosorbent assay (ELISA). Additionally, we focused on B-cell non-Hodgldn’s lymphoma (B-NHL) for survival analysis.

**Results:**

Influencing factors for ANA profile levels included age (ANA: *P* = 0.0035, SSA: *P* = 0.0553, SSB: *P* = 0.0025), gender (SSA: *P* = 0.0436), serum IgG (ANA, *P* = 0.0385; SSA, *P* = 0.0175; SSB, *P* = 0.0291), and erythrocyte sedimentation rate (ESR) (SSA: *P* = 0.0380). In subtype comparisons, ANA and SSB levels were significantly lower in low-grade B-NHL compared to Hodgkin lymphoma (HL) (low-grade B-NHL vs. NHL: ANA, *P* = 0.0107; SSB, *P* = 0.0126). Aggressive NHL exhibited a higher ANA profile compared to indolent NHL (aggressive NHL vs. indolent NHL: ANA, *P* = 0.0262; SSA, *P* = 0.0136; SSB, *P* = 0.0280). Kaplan–Meier analyses identified SSA and SSB as potential prognostic biomarkers in patients with B-NHL undergoing chemotherapy.

**Conclusion:**

Our study evaluated ANA profile in various subtypes of lymphoma and demonstrated the prognostic value of autoantibodies in predicting clinical outcomes. The results highlight the potential of incorporating ANA profile into the prognostic assessment of lymphoma.

**Supplementary Information:**

The online version contains supplementary material available at 10.1186/s12885-024-13198-2.

## Introduction

Antinuclear antibodies (ANA) represent a broad spectrum of autoantibodies that target components within the nucleus, such as DNA, RNA, proteins, or a molecular complex comprising these substances in the nucleus. The diverse array of ANA includes anti-chromatin antibodies (targeting double-stranded DNA and histones), anti-SM/RNP antibodies, anti-SSB/La antibodies (SSB), anti-SSA/Ro antibodies (SSA), anti-Scl-70 antibodies, anti-centromere antibodies, and anti-Jo-1 antibodies, and others. ANA profile served as a critical serological marker for diagnosing autoimmune diseases. Concurrently, there has been an increase in research reporting elevated ANA profile in patients with malignant tumors [1–3], implicating a potential association between ANA profile and the pathogenesis, progression, and prognosis of malignancies [4–6]. These results indicated a possible role for ANA profile as biomarkers for disease progression and patient outcomes.

Lymphoma is a class of malignant neoplasms originating from the lymphopoietic system, with hallmark clinical features including lymphadenopathy, splenomegaly, fever, nocturnal diaphoresis, inadvertent weight loss, and other systemic symptoms. Conventionally, lymphomas are categorized into Hodgkin’s lymphoma (HL), which constitutes approximately 10% of lymphoma cases, and non-Hodgkin lymphoma (NHL), making up the remaining 90% [7]. Further subclassification of NHL includes lymphoblastic lymphoma (LBL), B-cell non-Hodgldn’s lymphoma (B-NHL), and peripheral T-cell lymphoma (PTCL), each differing in cellular origin. Subtypes of NHL can be also divided into either aggressive or indolent, each presenting distinct pathogeneses and clinical manifestations [8]. The therapeutic regimen for NHL is tailored according to the specific subtype and stage of the disease [9].

The etiology of lymphoma is intricately linked with autoimmunity. There is an increased incidence of lymphoma among patients with autoimmune diseases, and conversely, individuals with lymphoma are more susceptible to developing autoimmune conditions [10–13]. Serological autoantibodies (AAb) have shown significant potential as biomarkers for predicting the prognosis of lymphoma following chemotherapy [14] or immunotherapy interventions [15]. Specifically, ANA profile, which can be frequently detected in lymphoma cases [1], hold promise for diagnostic and prognostic applications. Research on diffuse large B-cell lymphoma (DLBCL) has indicated that ANA may serve as stage-independent prognostic factors, reflecting an effective immune response to the tumor [16]. Another investigation on lymphoma has also emphasized the importance of the ANA spectrum for predicting the stage and prognosis of lymphoma patients [17]. Despite these advances, the mechanisms underlying the production of ANA profile and its relationship with lymphoma remain elusive.

Serum autoantibody detection offers a convenient, rapid, and non-invasive method for assessment. Nonetheless, the predicting value of autoantibodies in lymphoma remains underappreciated, and analyses in different lymphoma pathological types are lacking. To elucidate serum ANA profile across various lymphoma subtypes and to explore potential value of these autoantibodies in predicting lymphoma outcomes, our research employed enzyme-linked immunosorbent assay (ELISA) for ANA profile detection. This study investigate the role of ANA in lymphoma and highlight the need for developing novel diagnostic and prognostic tools based on autoimmune serological responses.

## Materials and methods

### Patients characteristics and specimen collection

A total of 139 lymphoma blood samples were collected from the National Cancer Center/National Clinical Research Center for Cancer/Cancer Hospital from March 2012 to July 2019 before treatment. The inclusion criteria for patient selection were as follows: (1) a diagnosis of lymphoma confirmed via biopsy tissue examination; (2) a history of good health prior to the lymphoma diagnosis. The exclusion criteria included: (i) patients diagnosed with or suspected of having other malignant tumors; (ii) patients with acquired immune deficiency syndrome (AIDS) or in the active phase of autoimmune diseases, such as systemic lupus erythematosus (SLE), rheumatoid arthritis (RA), mixed connective tissue disease (MCTD), Sjögren’s Syndrome, and Scleroderma, among others. Among the patients included in the analysis, we identified one with rheumatoid arthritis, one with Crohn’s disease, and two with a history of psoriasis. Importantly, none were experiencing acute episodes of their autoimmune conditions, and their autoantibody levels were within the overall average range for the cohort (Figure S1). After sampling, blood samples were centrifuged at 4 °C for 10 min at 3000 rpm, after which the plasma was aliquoted into tubes and preserved at -80 °C.

Comprehensive patient data, encompassing age, gender, clinical diagnosis, and complete blood count data—including neutrophile granulocytes, lymphocytes, and monocytes—as well as platelet (PLT), lactate dehydrogenase (LDH) levels, erythrocyte sedimentation rates (ESR), C-reactive protein (CRP), albumin (ALB) indices, histological subtype, therapeutic regimen, and prognostic information were meticulously collected from electronic medical records. The conduct of this study was approved by the Medical Ethics Committee of the National Cancer Center/National Clinical Research Center for Cancer/Cancer Hospital, with informed consent secured from all participating patients.

### Detection of antinuclear antibodies (ANA)

The assessment of ANA levels in plasma samples was conducted utilizing the QUANTA Lite ANA ELISA kit (INOVA Diagnostics, Inc.). The assay involved a variety of antigens, such as chromatin (double-stranded DNA and histones), Sm/RNP, SS-A, SS-B, Scl-70, kinetochore, proliferation cell nuclear antigen (PCNA), and Jo-1, all of which were pre-coated on microporous strips. To facilitate the binding of ANA to these antigens, diluted blood samples alongside quality control samples were applied to the micropores. Post-washing, the strips were incubated with anti-human IgG-conjugated horseradish peroxidase. The subsequent addition of the substrate allowed for the quantification of enzyme activity through the detection of color intensity. Optical density (OD) values were measured at a wavelength of 450 nm employing the Thermo Scientific Microplate Reader. The calculation of sample values (in units) was performed as follows: the ratio of the sample’s OD value to that of the low positive quality control, multiplied by the unit value of the low positive quality control.

### Detection of anti-SS-A antibodies (SSA)

The QUANTA Lite SS-A ELISA kit (INOVA Diagnostics, Inc.) was used for the determination of anti-SS-A antibodies (total SSA antibodies, including both the 52 kDa and 60 kDa subunits) in plasma samples. Procedures for the assay and calculation of results were strictly adhered to the manufacturer’s protocol as described in “Detection of antinuclear antibodies (ANA)” section.

### Detection of anti-SS-B antibodies (SSB)

Similarly, the QUANTA Lite SS-B ELISA kit (INOVA Diagnostics, Inc.) was employed to measure the concentration of anti-SS-B antibodies in plasma samples. Both the execution of the assay and the computation of results were performed in rigid compliance with the guidelines provided by the kit, as outlined in “Detection of antinuclear antibodies (ANA)” section.

### Quality control

Low positive, high positive, and negative controls were implemented on each assay plate as quality control measures to ascertain the reliability of the kit and the accuracy of the experimental procedures. A plate was deemed valid if it met the following predetermined criteria: (1) the OD value of the high positive control exceeded that of the low positive control, which in turn was greater than that of the negative control; (2) the OD value of the high positive control was greater than 1; (3) the OD value of the negative control was less than or equal to 0.2; (4) the OD value of the low positive control was greater than double the OD of the negative control, or exceeded 0.25. Inter-assay variability was evaluated by testing nine randomly selected samples on each plate (six plates in total), followed by the calculation of the coefficient of variation (CV), ensuring it remained below 20% (Figure S2).

### Statistical analysis

Normality tests were conducted before commencing data analysis. Normally distributed data were expressed as the mean ± standard deviation (SD), whereas non-normally distributed data were presented using the median and interquartile range [P_25_, P_75_]. The Mann–Whitney U test and Student’s t-test were applied to analyze continuous variables that were non-normally and normally distributed, respectively. For multiple group comparisons, the Kruskal–Wallis Rank Sum Test, supplemented by Dunn’s multiple comparisons test, was utilized. Correlation analyses were performed using Pearson’s correlation coefficient. Survival curves were constructed using Kaplan–Meier (K-M) analyses and p-values were calculated by Log-rank test. All statistical analyses and graphical representations were carried out using IBM SPSS Statistics (version 25.0), GraphPad Prism (version 9.0), and the statistical software R (version 4.2.3). A *p*-value < 0.05 was considered statistically significant.

## Results

### Baseline demographics and clinical characteristics

A total of 139 lymphoma patients were included in this study, comprising 17 cases of Hodgkin lymphoma (HL) and 122 cases of non-Hodgkin lymphoma (NHL). The NHL cohort consisted of 13 lymphoblastic lymphomas (LBL), 60 B-cell NHLs (B-NHL), and 49 peripheral T-cell lymphomas (PTCL). The LBL category included 2 B-cell lymphoblastic lymphomas (B-LBL) and 11 T-cell lymphoblastic lymphomas (T-LBL). The B-NHL category encompassed 13 diffuse large B-cell lymphoma (DLBCL), 11 follicular lymphomas (FL), 13 mantle cell lymphomas (MCL), 11 mucosa-associated lymphoid tissue lymphomas/marginal zone lymphomas (MALT-MZL), 6 chronic lymphocytic leukemias/small lymphocytic lymphomas (CLL/SLL), and 6 Burkitt’s lymphomas (BL). The PTCL category comprised 14 extranodal NK/T-cell lymphomas (ENKTL), 11 angioimmunoblastic T-cell lymphomas (AITL), 10 anaplastic large-cell lymphomas (ALCL), 10 peripheral T-cell lymphomas, not otherwise specified (PTCL-NOS), and 4 cutaneous T-cell lymphomas (CTCL) (Table 1). Histopathological evaluation of biopsy samples provided diagnoses for each lymphoma subtype. CLL/SLL, MALT-MZL, and FL were classified as indolent NHL, whereas PTCL, BL, and DLBCL were categorized as aggressive NHL. Notably, all MCL cases were identified as nodal MCL with symptomatic bulky nodal or extra-nodal disease [18], and therefore, categorized as aggressive NHL. Besides, one patient with FL that transformed into DLBCL and a patient with MALT-MZL that transformed into DLBCL are considered to have aggressive NHL. The demographics and clinical characteristics of the subtypes of lymphoma are showed in Table S1. The demographics and clinical characteristics of the aggressive and indolent NHL are delineated in Table S2.

**Table 1:** Pathological subtypes of the cohort

Pathological Type	Number
**Hodgkin’s lymphoma (HL)**	17
**non-Hodgkin’s lymphoma (NHL)**	122
** B-cell non-Hodgldn’s lymphoma (B-NHL)**	60
** Burkitt’s lymphoma (BL)**	6
** follicular lymphoma (FL)**	11
** chronic lymphocytic leukemia/small lymphocytic lymphoma (CLL/SLL)**	6
** diffuse large B cell lymphoma (DLBCL)**	13
** mucosa-asociated lymphoid tissue-marginal zone lymphoma (MALT-MZL)**	11
** mantle cell lymphoma (MCL)**	13
** peripheral T-cell lymphom (PTCL)**	49
** anaplastic large-cell lymphoma (ALCL)**	10
** extranodal NK/T-cell lymphoma (ENKTL)**	14
** angioimmunoblastic T-cell lymphoma (AITL)**	11
** peripheral T-cell lymphoma, unspecified (PTCL-NOS)**	10
** cutaneous T-cell lymphoma (CTCL)**	4
** lymphoblastic lymphoma (LBL)**	13
** T-cell lmphoblastic lymphoma (T-LBL)**	11
** B-cell lmphoblastic lymphoma (B-LBL)**	2

### Determinants associated with antinuclear antibodies

**Fig. 1:**
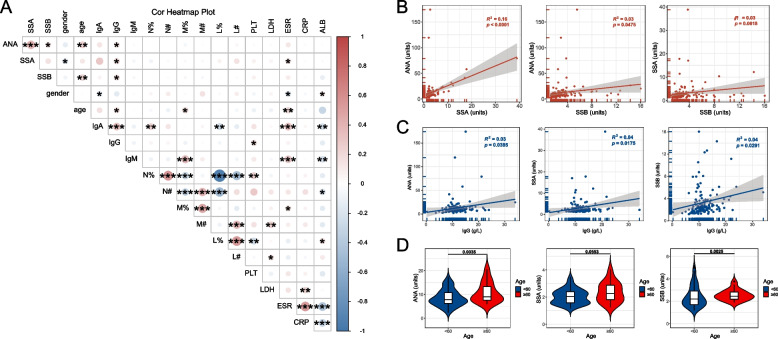
Determinants associated with antinuclear antibodies. **A** Correlation heatmap of the ANA profile levels, clinical characteristics, and laboratory parameters. Pearson’s r correlation method. **B** Simple linear regression of ANA and SSA, ANA and SSB, as well as SSA and SSB. **C** Simple linear regression of ANA, SSA, SSB, and IgG. **D** Comparison of ANA profile between patients under and over 60 years of age. Mann–Whitney U-test. Levels of significance: *: *P* < 0.05; **: *P* < 0.01; ***: *P* < 0.001; ****: *P* < 0.0001

A heatmap depicted the expression profile of antibodies in patients (Figure S3). Pearson’s correlation analysis was performed to identify potential factors associated with autoantibodies (Fig. 1A). Significant correlations were observed between ANA and SSA, as well as between ANA and SSB (ANA vs. SSA, r^2^ = 0.16, *P* < 0.0001; ANA vs. SSB, r^2^ = 0.03, *P* = 0.0475). The correlation between SSA and SSB approached significance but was not statistically significant (r^2^ = 0.03, *P* = 0.0618) (Fig. 1B). Additionally, plasma IgG levels were significantly associated with all three autoantibodies (ANA, r^2^ = 0.03, *P* = 0.0385; SSA, r^2^ = 0.04, *P* = 0.0175; SSB, r^2^ = 0.04, *P* = 0.0291) (Fig. 1C). Furthermore, our analysis revealed age, gender, and ESR as potential influencing factors for autoantibodies. A higher ANA profile was observed in patients over 60 years of age, with statistically significant results for ANA (*P* = 0.0035) and SSB (*P* = 0.0025), while SSA showed a trend towards significance (*P* = 0.0553) (Fig. 1D). Female patients exhibited higher levels of autoantibodies, with SSA reaching statistical significance (*P* = 0.0436) and SSB approaching significance (*P* = 0.0849) (Figure S4A). ESR was found to be statistically associated with SSA levels (r^2^ = 0.03, *P* = 0.0380) (Figure S5B).

### ANA profile in different histological lymphoma subtypes

**Fig. 2:**
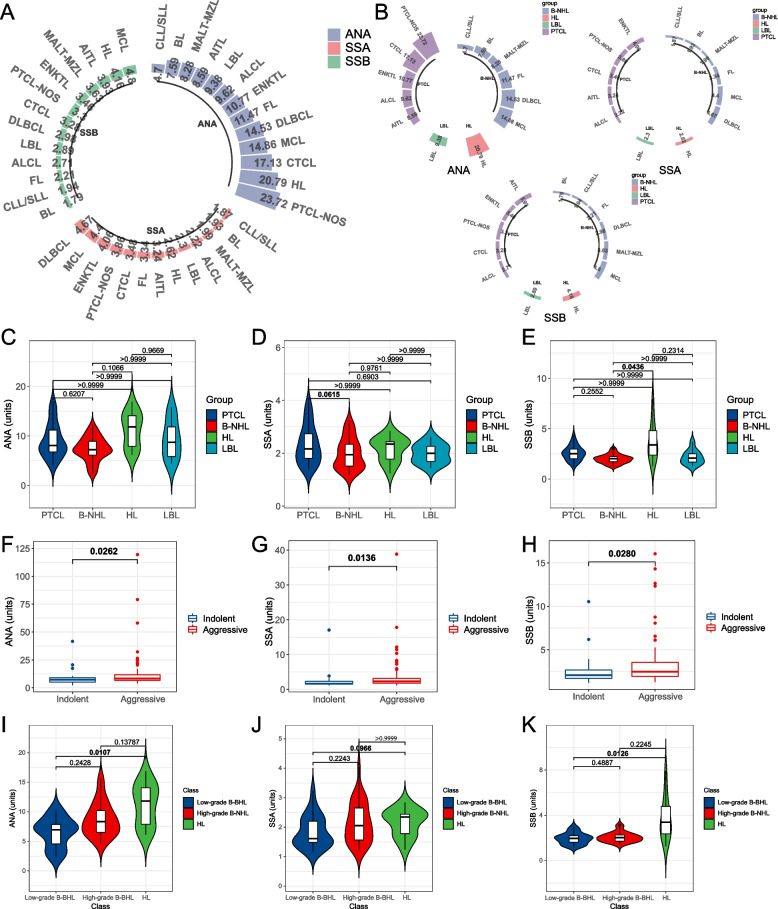
Comparison of ANA profile between different lymphoma subtypes. **A** Circular bar chart depicting the levels of ANA, SSA, and SSB across different pathological subtypes. The numbers represent the average expression levels for each lymphoma subtype. **B** ANA, SSA, and SSB levels in different pathological subtypes, respectively. The numbers represent the average expression levels for each lymphoma subtype. **C**-**E** Comparisons of ANA, SSA, and SSB between B-NHL, LBL, PTCL, and HL. Kruskal–Wallis Rank Sum Test with Dunn’s multiple comparisons test. **F**–**H** Comparisons of ANA, SSA, and SSB between aggressive and indolent NHL. Mann–Whitney U-test. **I**-**K** Comparisons of ANA, SSA, and SSB between low-grade B-BHL, high-grade B-BHL, and HL. Kruskal–Wallis Rank Sum Test with Dunn’s multiple comparisons test

We assessed autoantibody levels across various histological lymphoma subtypes. A circular bar chart illustrated the expression levels of the three autoantibodies among the subtypes (Fig. 2A). The top five lymphoma subtypes for serum ANA expression were PTCL-NOS, HL, CTCL, MCL, and DLBCL. In terms of SSA expression, the leading subtypes were DLBCL, MCL, ENKTL, PTCL-NOS, and CTCL. With regard to SSB expression, the foremost subtypes were MCL, HL, AITL, MALT-MZL, and ENKTL. These subtypes were further categorized into B-NHL, PTCL, LBL, and HL, with detailed rankings provided in Fig. 2B. Multiple comparison analyses indicated that B-NHL had significantly lower levels of SSB compared to HL (SSB: B-NHL vs. HL, 2.14 units vs. 3.50 units, *P* = 0.0436) (Fig. 2E) (Table S3). And B-NHL showed marginally lower levels of SSA when compared to PTCL (SSA: B-NHL vs. PTCL, 2.05 units vs. 2.51 units, *P* = 0.0615) (Fig. 2D) (Table S3). Comparing NHL with HL, ANA, and SSB were significantly elevated in HL (HL vs. NHL, ANA: 12.26 units vs. 8.29 units, *P* = 0.0480; SSB: 3.50 units vs. 2.39 units, *P* = 0.0234), whereas SSA levels, though higher in HL, did not reach statistical significance (HL vs, NHL, 2.38 units vs. 2.16 units, *P* = 0.4798) (Figure S5) (Table S3).

### ANA profile in aggressive and indolent NHL

Autoantibody levels were compared between aggressive and indolent NHL, revealing a clear association with the invasiveness of lymphoma. Aggressive NHL displayed higher levels of autoantibodies compared to indolent NHL (aggressive vs. indolent NHL: ANA, 8.37 units vs. 7.21 units, *P* = 0.0262; SSA, 2.31 units vs. 1.64 units, *P* = 0.0136; SSB, 2.46 units vs. 2.07 units, *P* = 0.0280) (Fig. 2F-H) (Table S3). Apart from ANA profile, significant differences were noted in laboratory indices such as serum IgA, neutrophil granulocytes, lymphocytes, ESR, CRP, and ALB between aggressive and indolent NHL (Table S2). Besides, we specifically focused on B cell lymphoma. We compared ANA profile among low-grade B-NHL (including CLL/SLL, MALT-MZL, and FL), high-grade B-NHL (including MCL, BL, and DLBCL) and HL. Our results indicate that the ANA profile in HL was significantly higher than that in low-grade B-NHL, with statistically significant differences observed for ANA (*P* = 0.0107) and SSB (*P* = 0.0126) (Fig. 2I and K). Additionally, SSA demonstrated a trend towards significance (*P* = 0.0966) (Fig. 2J). However, we did not observe a statistically significant difference in ANA profiles between low-grade B-NHL and high-grade B-NHL.

### Autoantibodies as potential prognostic biomarkers

**Fig. 3:**
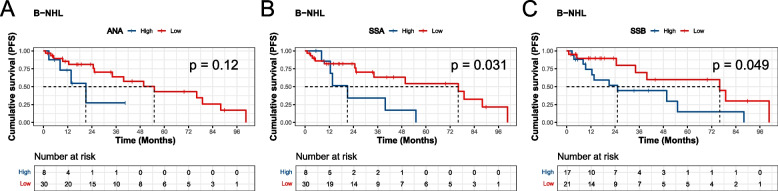
ANA profile as potential prognostic biomarkers. **A**-**C** Kaplan–Meier (K-M) survival curve of progression free survival (PFS) in B-NHL patients receiving chemotherapy. K-M analysis was performed using the R package “survminer” to determine the optimal cut-off values for categorizing high and low biomarker levels based on maximizing the log-rank test statistic

Subsequently, we investigated the ANA profile as a prognostic biomarker in lymphoma. Given the diverse range of lymphoma subtypes and treatment regimens included, we focused specifically on patients with B-NHL undergoing chemotherapy. We identified SSA and SSB as potential prognostic biomarkers in this cohort (Fig. 3A-C). K-M analysis was performed using the R package “survminer” to determine the optimal cut-off values (Threshold: ANA, 10.97 units; SSA, 2.93 units; SSB, 2.22 units) for categorizing high and low biomarker levels based on maximizing the log-rank test statistic. Patients with low SSA levels exhibited significantly longer progression-free survival (PFS) compared to those with high SSA levels (High-SSA vs. Low-SSA: 21.00 vs. 76.03 months, *P* = 0.031) (Fig. 3B). Similarly, the low SSB group demonstrated longer PFS compared to the high SSB group (High-SSB vs. Low-SSB: 25.20 vs. 76.03 months, *P* = 0.049) (Fig. 3C). These findings suggested that autoantibodies may serve as promising prognostic biomarkers in lymphoma.

## Discussion

AAb have demonstrated efficacy as biomarkers for prognosis prediction in lymphoma. Despite ANA profile being among the earliest identified and the most commonly utilized autoantibodies, the predictive role of ANA within lymphoma remains underreported. To address this gap, we assessed plasma samples from a diverse cohort of lymphoma patients, exploring the determinants of ANA levels and ANA profile across different lymphoma subtypes. Specifically, the investigation focused on the prognostic capacity of ANA profile.

AAb are antibodies formed against self-antigens present in tissues, organs, cells, and cell components. The mechanisms underlying AAb production are not fully elucidated, and the factors influencing their generation are multifaceted. Our study observed significant elevations in ANA profile levels among individuals over 60 years, aligning with findings from prior research [19]. We also observed that the level of serum SSA, a specific type of ANA which targeted the Ro60/Ro52 ribonucleoproteins (RNPs), was higher in female patients than in male patients. This is indicative of heightened immunoreactivity in females, leading to increased autoreactivity and contributing to the sex-based prevalence observed in autoimmune diseases [20–22]. ESR has been frequently identified as a risk factor for autoimmune conditions [23] and is valued as an inflammatory marker reflective of disease activity [24]. Consistent with earlier studies, our results suggest that ESR may correlate with autoantibody levels, further establishing the complex interplay between inflammation and autoimmunity.

We observed that the levels of ANA profile were higher in patients with HL compared to those with NHL. Specifically, low-grade B-NHL exhibited significantly lower autoantibody levels than HL. In contrast, autoantibody levels in high-grade B-NHL did not show significant differences when compared to HL. Both HL and many subtypes of high-grade B-NHL are often associated with viral infections and immune dysregulation. Various autoimmune diseases, including systemic lupus erythematosus (SLE) and Sjögren’s syndrome, have been linked to the development of these lymphomas [25–27]. In contrast, low-grade B-NHL, such as FL, did not exhibit a strong association with these autoimmune factors. This may explain the significant differences in autoantibody levels between low-grade B-NHL and HL. In parallel research on different autoantibodies, Cil et al. found antineutrophil cytoplasmic antibodies (ANCA) to be present exclusively in HL patients, with no detectable ANCA positivities in NHL patients [28]. Another investigation into autoantibody prevalence within lymphoma cohorts disclosed that a minimum of one antiphospholipid antibody (APA) was positively identified in 26% of NHL patients and 38% of HL patients [29]. Nevertheless, certain studies have reported elevated levels of autoantibodies in NHL relative to HL [30, 31]. The variability may attribute to the distinct classes of autoantibodies and the methodologies employed for their detection. In the comparative analysis of lymphoma subtypes, specific pathological classes demonstrated higher ANA profile expressions. MCL ranked within the top five of lymphoma subtypes in terms of expression levels for the three antinuclear antibodies. Besides, PTCL-NOS, HL, CTCL, DLBCL, and ENKTL featured among the top five subtypes for expression of two out of the three evaluated antinuclear antibodies. These findings underscore the intricacies of autoantibody prevalence within varied lymphoma subtypes and highlight the necessity for further research in this domain.

NHL is a diverse category of diseases, which can be classified into aggressive subtypes, such as DLBCL, and indolent diseases, including CLL/SLL, MALT-MZL, and FL [8]. The choice of therapeutic strategy is contingent upon the specific lymphoma subtype. Santos et al. demonstrated that MIB-1 immunohistochemistry revealed a marked elevation in the proliferation index (PI) correlating with increased tumor aggressiveness [32]. Concurrently, Wang et al. recognized Ki67 as a significant marker capable of differentiating between aggressive and indolent lymphoma types [33]. MicroRNAs (miRNAs) have similarly been noted to provide insights into the degree of aggressiveness of lymphoma [34]. However, to date, there lacks an investigation into the linkage between autoantibodies and the aggressiveness of lymphoma. In our research, serum ANA profile in patients with aggressive NHL was apparently higher than those with indolent NHL. This suggests that ANA profile could serve as predictive markers for aggressive NHL, offering a valuable adjunct to pathological evaluation.

ANA profile had potential predictive roles in lymphoma [16]. In our research, we identified both SSB and SSA as significant prognostic biomarkers in patients with B-NHL undergoing chemotherapy. SSB, involved in essential processes of RNA biogenesis and function [35], was found to correlate with a less favorable prognosis, characterized by shorter PFS in patients with elevated titers. Additionally, patients with high levels of SSA also demonstrated similarly poor prognostic outcomes. These findings indicate that the ANA profile holds prognostic value in predicting clinical outcomes for patients with lymphoma. Integrating the ANA profile into current prognostic models has the potential to enhance the precision of risk stratification and improve personalized treatment regimens. Nonetheless, further longitudinal studies with larger cohorts are essential to validate our preliminary findings and to assess whether ANA profile can serve as reliable biomarkers for disease progression and patient prognosis.

The strength of our study was a cohort encompassing various subtypes of lymphoma, an aspect that was absent in prior autoantibody research. Moreover, we implemented stringent quality control measures, ensuring that the CV for samples across different plates was consistently maintained at less than 20%. Additionally, our investigation probed the role of ANA profile as a prognostic biomarker in lymphoma. However, there were several limitations. Firstly, our analysis was confined to ANA profile in lymphoma patients without the inclusion of a healthy control group for comparison. Secondly, it is crucial to verify these findings within a larger cohort of lymphoma subtypes. Thirdly, the biological mechanisms underlying the produce of autoantibodies in lymphoma patients remain unclear and warrant further in-depth investigation. Given the potential of autoantibodies as biomarkers, it is important to conduct further studies to investigate the diagnostic and prognostic role of ANA profile in lymphoma as well as other malignant tumors.

In conclusion, our research investigated the effectiveness of plasma autoantibodies as biomarkers in lymphoma. Our analysis identified age, gender, serum IgG, and ESR as significant factors associated with ANA profile. Furthermore, we assessed ANA profile across various pathological subtypes of lymphoma and illustrated their predictive significance concerning the outcomes of lymphoma.

## Supplementary Information

Supplementary Material 1.

## Data Availability

The data that support the findings of this study are available from the corresponding author upon reasonable request.

## Abbreviations

ALC: Absolute lymphocyte count
AIDS: Acquired immune deficiency syndrome
ALB: Albumin
ALCL: Anaplastic large-cell lymphoma
AITL: Angioimmunoblastic T-cell lymphoma
SSA: Anti-SSA/Ro antibodies
SSB: Anti-SSB/La antibodies
ANCA: Antineutrophil cytoplasmic antibodies
ANA: Antinuclear antibodies
APA: Antiphospholipid antibody
AAb: Autoantibodies
B-NHL: B-cell non-Hodgldn’s lymphoma
B-LBL: B-cell lmphoblastic lymphoma
BL: Burkitt’s lymphoma
CRP: C-reactive protein
CLL/SLL: Chronic lymphocytic leukemia/small lymphocytic lymphoma
CV: Coefficient of variation
CTCL: Cutaneous T-cell lymphoma
DLBCL: Diffuse large B cell lymphoma
ELISA: Enzyme-linked immunosorbent assay
ESR: Erythrocyte sedimentation rate
ENKTL: Extranodal NK/T-cell lymphoma
FL: Follicular lymphoma
HL: Hodgkin’s lymphoma
K-M: Kaplan-Meier
LDH: Lactate dehydrogenase
LBL: Lymphoblastic lymphoma
MCL: Mantle cell lymphoma
miRNAs: MicroRNAs
MALT-MZL: Mucosa-asociated lymphoid tissue-marginal zone lymphoma
NHL: Non-Hodgkin’s lymphoma
OD: Optical density
PTCL: Peripheral T-cell lymphoma
PTCL-NOS: Peripheral T-cell lymphoma, unspecified
PLT: Platelet
PFS: Progression-free survival
PCNA: Proliferation cell nuclear antigen
PI: Proliferation index
SD: Standard deviation
T-LBL: T-cell lmphoblastic lymphoma
