# Supplementary material for: Evaluating plasma antinuclear autoantibody profile as a prognostic biomarker in lymphoma
Source: BMC Cancer. 2024 Nov 26;24:1451. doi: 10.1186/s12885-024-13198-2 (PMC11590230; doi:10.1186/s12885-024-13198-2)
Supplement: Supplementary file 1 — Supplementary Material 1. [file 12885_2024_13198_MOESM1_ESM.docx]

**Supplementary materials for**

**Evaluating Plasma Antinuclear Autoantibody Profile as Prognostic Biomarkers in Lymphoma**

**The PDF file includes:**

Figures S1 to S5

Tables S1 to S3

**Supplementary figure 1**

**
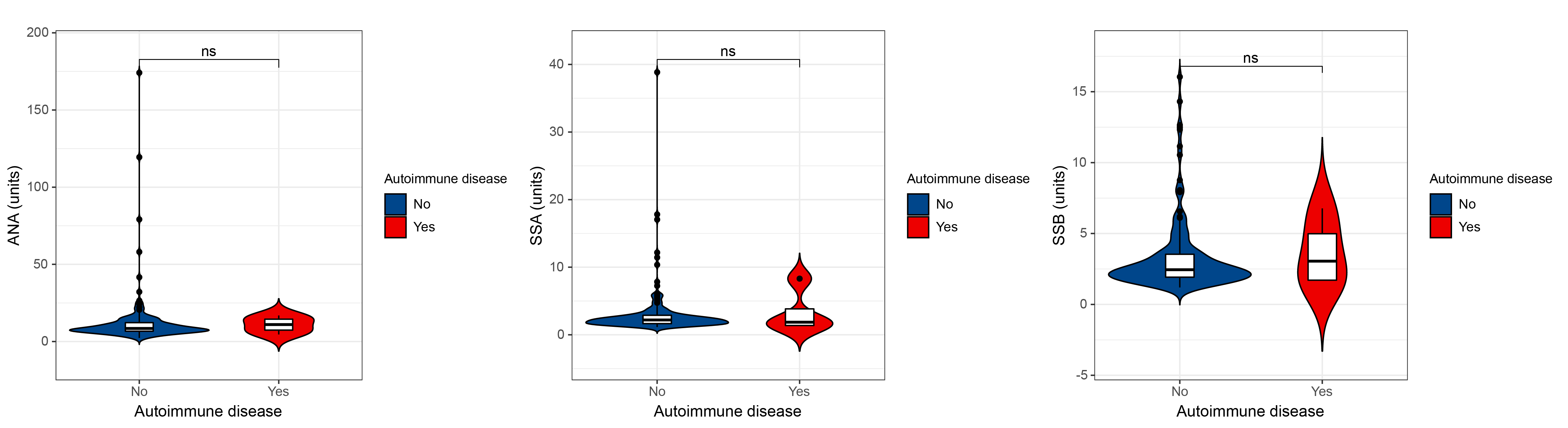
**

**Fig.S1. Comparison of ANA profiles between patients with or without a history of autoimmune diseases.**

**Supplementary figure 2**

**
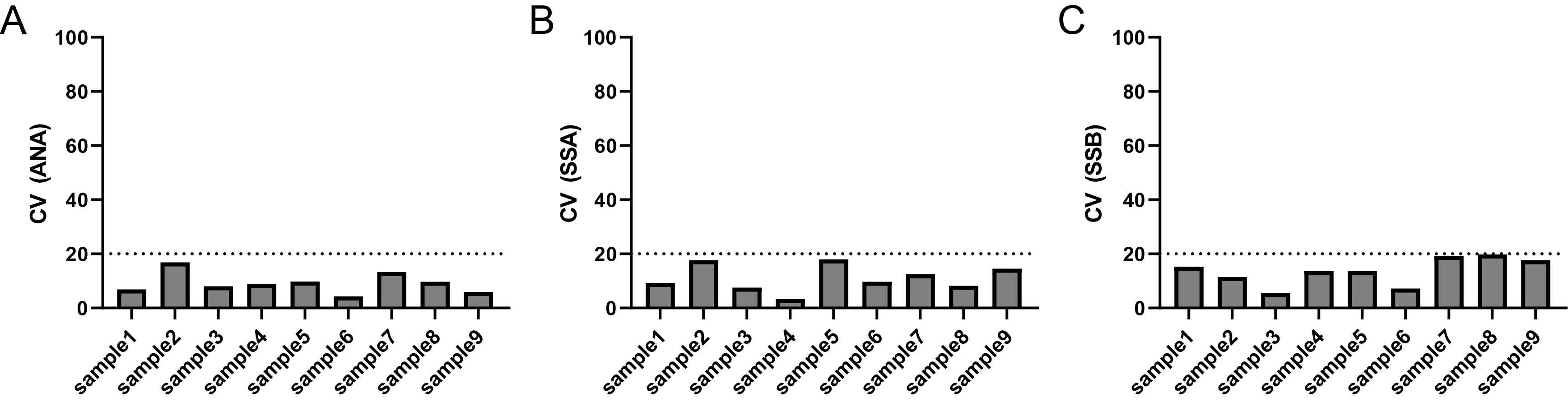
**

**Fig.S2. Quality control of the experiment.** (A) Coefficient of variation (CV) of ANA in different plate. (B) CV of SSA in different plate.(C) CV of SSB in different plate.

**Supplementary figure 3**


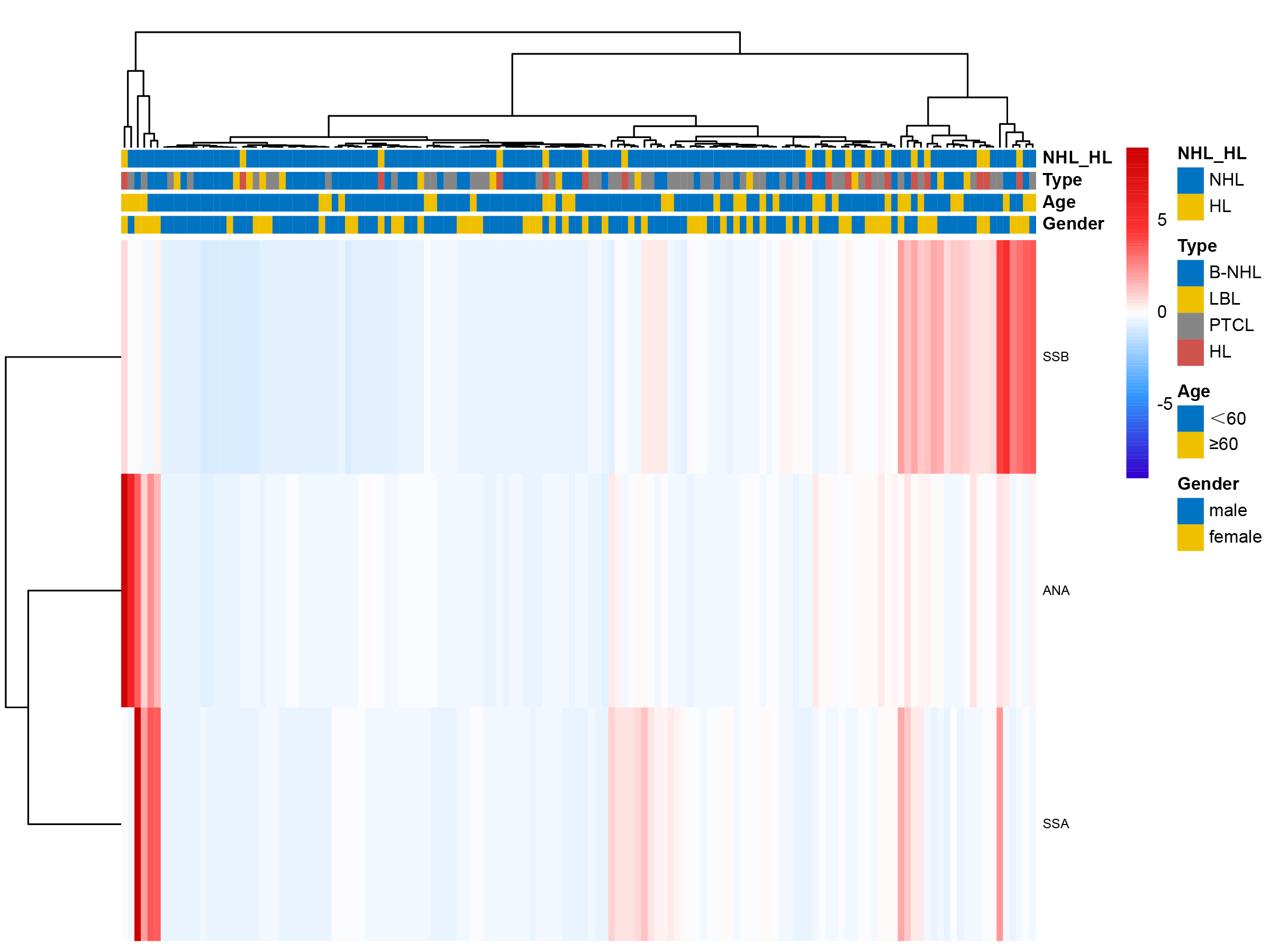


**Fig.S3.** **Heatmap of the ANA profile.**

**Supplementary figure 4**

**
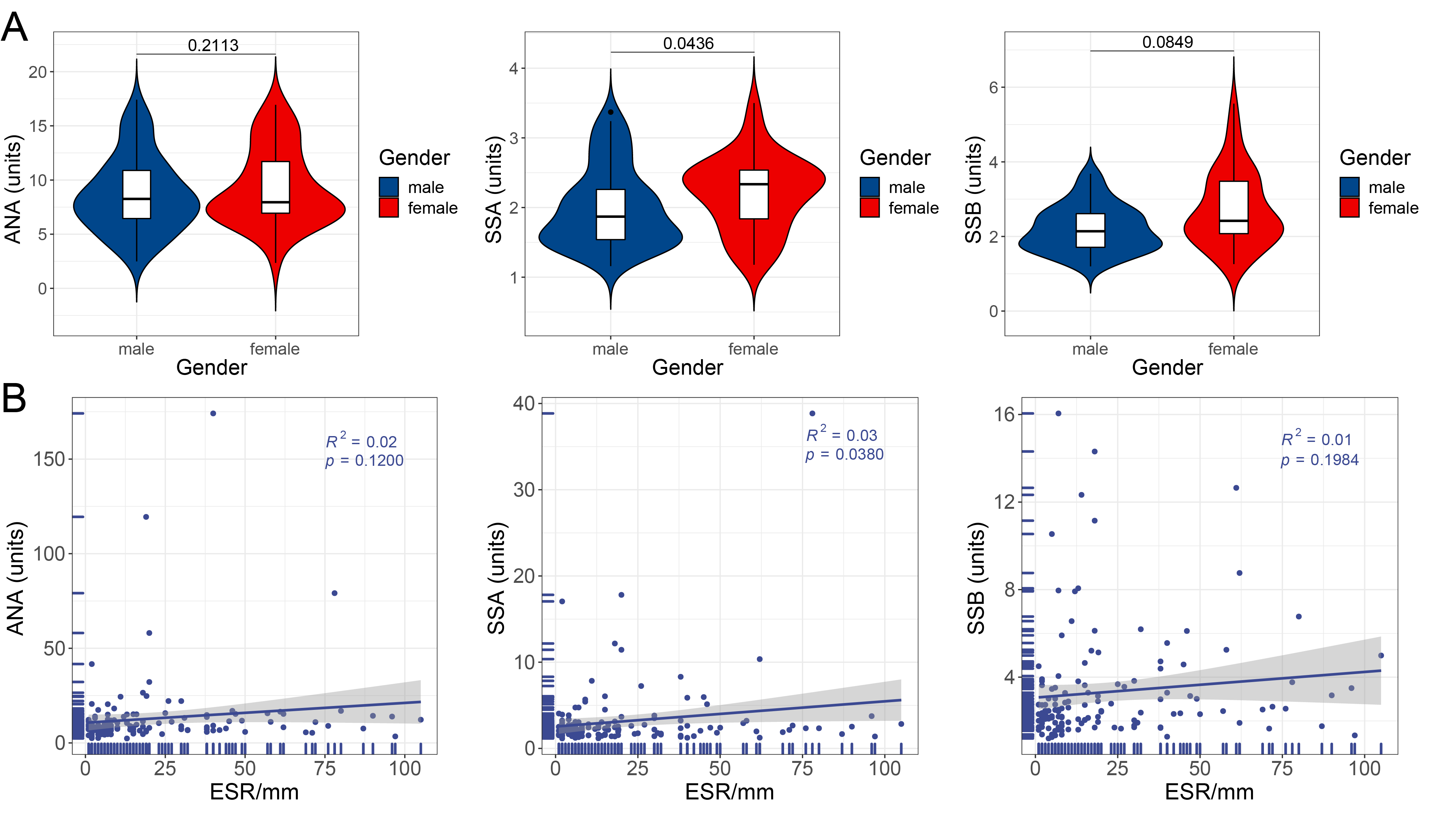
**

**Fig.S4. Association between gender, ESR and antinuclear antibodies profiles.** (A) Auto-antibodies comparison between different genders. (B) Simple linear regression of auto-antibodies and ESR.

**Supplementary figure 5**

**
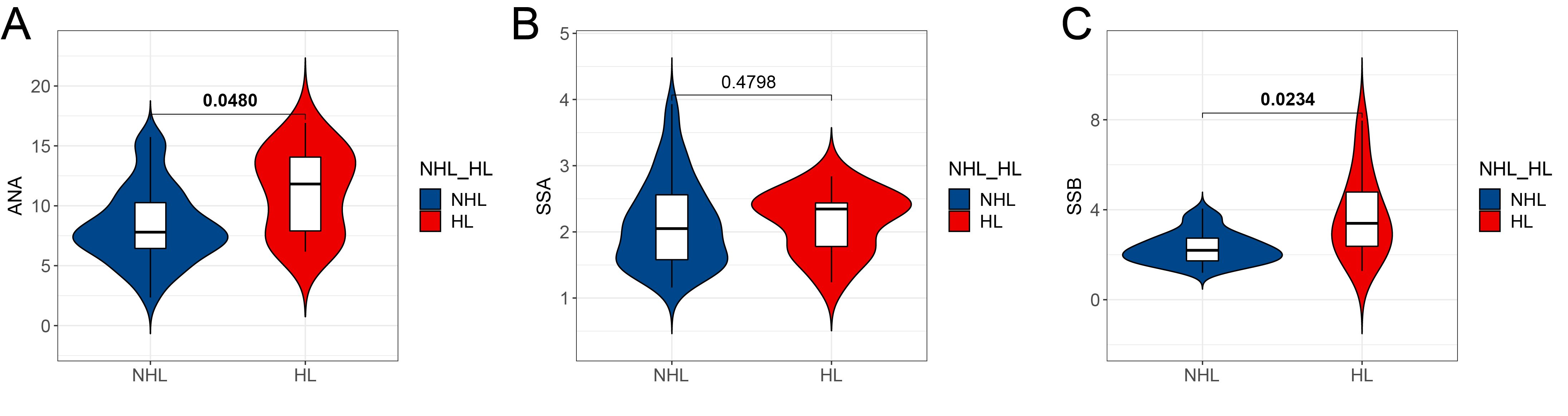
**

**Fig.S5. ANA profile comparison between NHL and HL.** **(A)-(C)** ANA, SSA, and SSB comparison between NHL and HL. Mann-Whitney U-test.

**Table S1. Demographics and clinical characterization of the subtypes of lymphoma**

| **Characteristic** | **HL** | **B-NHL** | **PTCL** | **LBL** |
| --- | --- | --- | --- | --- |
| **Sample size, n** | 17 | 60 | 49 | 13 |
| **Gender** |  |  |  |  |
| **Male** | 7 (41%) | 38 (63%) | 30 (61%) | 10 (77%) |
| **Female** | 10 (59%) | 22 (37%) | 19 (39%) | 3 (23%) |
| **Age (year)** | 38.41±18.45 | 51.10±14.88 | 48.90±14.59 | 26.46±12.59 |
| **IgA (g/L)** | 2.61±1.25 | 2.09±1.31 | 2.72±1.47 | 1.86±0.78 |
| **IgG (g/L)** | 12.55±3.81 | 11.76±4.00 | 12.14±4.60 | 10.17±3.42 |
| **IgM (g/L)** | 1.66±1.65 | 2.19±7.68 | 1.32±0.89 | 0.91±0.64 |
| **Neutrophile granulocyte (%)** | 65.90±16.87 | 57.65±15.98 | 63.67±13.48 | 62.43±23.21 |
| **ANC(10^9^/L)** | 4.82±2.87 | 3.71±2.14 | 4.60±2.88 | 4.41±2.76 |
| **Lymphocyte** **(%)** | 22.94±13.74 | 31.49±15.29 | 25.64±11.91 | 28.21±23.53 |
| **ALC (10^9^/L)** | 1.50±0.78 | 2.08±2.28 | 1.74±1.66 | 1.44±0.70 |
| **Monocyte (%)** | 6.89±2.93 | 8.24±4.27 | 8.21±4.58 | 6.99±5.21 |
| **AMC (10^9^/L)** | 0.49±0.28 | 0.49±0.26 | 0.53±0.32 | 0.42±0.32 |
| **PLT (10^9^/L)** | 254.82±103.06 | 232.05±92.82 | 235.47±89.66 | 247.31±77.92 |
| **LDH (U/L)** | 206.06±57.65 | 210.62±83.60 | 247.33±190.74 | 314.92±260.68 |
| **ESR (mm/h)** | 32.00[7.00,60.00] | 8.00[3.25,18.75] | 14.00[6.50,34.00] | 15.00[7.00,37.50] |
| **CRP (mg/L)** | 0.30[0.03,3.66] | 0.19[0.01,1.73] | 0.73[0.10,1.54] | 0.44[0.04,3.39] |
| **ALB (g/L)** | 43.61±5.90 | 44.10±4.82 | 42.66±4.73 | 44.70±4.75 |
| **Therapy** |  |  |  |  |
| **Chemotherapy** | 8 (47%) | 35 (58%) | 28 (57%) | 9 (69%) |
| **Chemoradiotherapy** | 6 (35%) | 10 (17%) | 13 (27%) | 2 (15%) |
| **Radiotherapy** | 1 (6%) | 3 (5%) | 3 (6.1%) | 0 (0%) |
| **Other** | 1 (6%) | 7 (12%) | 4 (8.2%) | 1 (7.7%) |
| **Unknown** | 1 (6%) | 5 (8%) | 1 (2.0%) | 1 (7.7%) |
| **Abbreviations:** Hodgkin's lymphoma, HL; B-cell non-Hodgldn's lymphoma, B-NHL; peripheral T-cell lymphom, PTCL; lymphoblastic lymphoma, LBL; absolute neutrophil count, ANC; absolute lymphocyte count, ALC; absolute monocyte count, AMC; platelet, PLT; lactate dehydrogenase, LDH; [erythrocyte sedimentation rate](javascript:;), ESR; C-reactive protein, CRP; [albumin](javascript:;), ALB. | | | | |

**Table S2. Demographics and clinical characterization of aggressive and indolent lymphoma.**

| **Characteristic** | **Aggressive** | **Indolent** | **P value** |
| --- | --- | --- | --- |
| **Sample size, n** | 79 | 26 |  |
| **Gender** |  |  |  |
| **Male** | 49 (62%) | 16 (62%) | 0.965 |
| **Female** | 30 (38%) | 10 (39%) |  |
| **Age(year)** | 50.44±15.49 | 49.65±12.61 | 0.815 |
| **IgA (g/L)** | 2.64±1.53 | 1.62±0.69 | **<0.001** |
| **IgG (g/L)** | 12.43±4.56 | 10.61±3.31 | 0.064 |
| **IgM (g/L)** | 1.18±0.83 | 3.66±11.4 | 0.279 |
| **Neutrophile granulocyte (%)** | 63.28±13.34 | 51.78±17.47 | **<0.001** |
| **ANC(10^9^/L)** | 4.50±2.65 | 2.98±1.80 | **0.008** |
| **Lymphocyte (%)** | 25.62±11.26 | 35.67±17.63 | **<0.001** |
| **ALC (10^9^/L)** | 1.69±1.51 | 2.58±3.56 | **0.044** |
| **Monocyte (%)** | 8.03±4.23 | 8.60±5.12 | 0.572 |
| **AMC (10^9^/L)** | 0.52±0.30 | 0.45±0.22 | 0.286 |
| **PLT (10^9^/L)** | 236.97±97.71 | 241.27±77.47 | 0.899 |
| **LDH (U/L)** | 229.03±107.94 | 191.68±61.00 | 0.126 |
| **ESR (mm/h)** | 10.00[6.00,20.00] | 5.50[2.00,12.25] | **0.002** |
| **CRP (mg/L)** | 0.73[0.04,1.84] | 0.14[0.00,0.83] | **0.032** |
| **ALB (g/L)** | 42.83±4.87 | 45.28±4.43 | **0.025** |
| **Therapy** |  |  | 0.606 |
| **Chemotherapy** | 46 (58%) | 15 (58%) |  |
| **Chemoradiotherapy** | 17 (22%) | 6 (23%) |  |
| **Radiotherapy** | 3 (4%) | 2 (8%) |  |
| **Other** | 3 (4%) | 2 (8%) |  |
| **Unknown** | 10 (13%) | 1 (4%) |  |
| **Abbreviations:** absolute neutrophil count, ANC; absolute lymphocyte count, ALC; absolute monocyte count, AMC; platelet, PLT; lactate dehydrogenase, LDH; [erythrocyte sedimentation rate](javascript:;), ESR; C-reactive protein, CRP; [albumin](javascript:;), ALB. | | | |

**Table S3. Autoantibody profiles of different lymphoma [subtype](javascript:;)s.**

| **[Subtype](javascript:;)** | | **ANA** | **SSA** | **SSB** |
| --- | --- | --- | --- | --- |
| **[Pathological Type](javascript:;)** | **HL** | 12.26[7.70,14.53] | 2.38[1.79,2.74] | 3.50[2.37,5.28] |
|  | **NHL** | 8.29[6.60,11.77] | 2.16[1.61,2.96] | 2.39[1.89,3.33] |
|  | **B-NHL** | 7.83[6.48,10.40] | 2.05[1.54,2.71] | 2.14[1.72,3.11] |
|  | **PTCL** | 8.86[7.06,14.40] | 2.51[1.88,3.74] | 2.65[2.16,3.51] |
|  | **LBL** | 8.72[5.57,12.84] | 2.11[1.68,2.34] | 2.30[1.67,3.23] |
| **Aggressive or Indolent** | **Aggressive** | 8.37[6.71,11.70] | 2.31[1.80,3.17] | 2.46[1.94,3.54] |
|  | **Indolent** | 7.21[5.15,9.03] | 1.64[1.51,2.30] | 2.07[1.71,2.66] |
